# Utilizing Indonesian Empty Palm Fruit Bunches: Biochar Synthesis via Temperatures Dependent Pyrolysis

**DOI:** 10.3390/nano15010050

**Published:** 2024-12-31

**Authors:** Fairuz Gianirfan Nugroho, Abu Saad Ansari, Nurul Taufiqu Rochman, Shubhangi Satish Khadtare, Vijaya Gopalan Sree, Nabeen K. Shrestha, Afina Faza Hafiyyan, Hyunsik Im, Abu Talha Aqueel Ahmed

**Affiliations:** 1 Center of Excellence Applied Nanotechnology, Nano Center Indonesia, Puspiptek, South Tangerang 15314, Banten, Indonesia; fairuz.gianirfan@gmail.com (F.G.N.); saad@nano.or.id (A.S.A.);; 2 Research Center for Advanced Material, National Research and Innovation Agency (BRIN), Puspiptek, South Tangerang 15314, Banten, Indonesia; nurul@nano.or.id; 3 Department of Electronic Engineering, Hanyang University, Seoul 04763, Republic of Korea; 4 Division of Physics and Semiconductor, Dongguk University, Seoul 04620, Republic of Korea

**Keywords:** biochar, pyrolysis, empty fruit bunches, Raman spectroscopy, palm fruit

## Abstract

Biomass, though a major energy source, remains underutilized. Biochar from biomass pyrolysis, with its high porosity and surface area, is especially useful as catalyst support, enhancing catalytic activity and reducing electron recombination in photocatalysis. Indonesia, the world’s top palm oil producer, generated around 12 million tons of empty fruit bunches (EFBs) in 2023, making EFBs a promising biochar source. This study synthesizes biochar from leftover EFB fibers at 500, 800, and 1000 °C, analyzing structural changes via infrared and Raman spectroscopy, along with particle size and surface area analysis, laying the groundwork for future biochar research. The smallest particle size and highest surface area gained was 71.1 nm and 10.6 × 10^2^ m^2^/g. Spectroscopic analysis indicates that biochar produced at 1000 °C has produced nano-crystalline graphite with a crystallite size of approximately 5.47 nm. This provides higher defect density, although with lower conductivity. Other studies indicate that our biochar can be used as catalyst support for various green energy-related applications, i.e., counter electrodes, electrocatalysts, and photocatalysts.

## 1. Introduction

The management of biomass waste has become an increasingly pressing concern for countries worldwide, given the growing environmental and sustainability challenges posed by such waste. Biomass, a renewable energy resource derived from organic materials, is one of the most abundant resources on the planet. Despite its vast availability and potential, the utilization of biomass remains in the developmental stage, with continuous research dedicated to unlocking its full capabilities and addressing technological barriers [1]. Over the years, several biomass-based materials have been extensively studied in various fields as emerging materials, which include biodegradable materials, catalysts, adsorbents, fuel cells, solar cells, sensors, semiconductors, 2D materials, supercapacitors, electrode material for batteries (Li-ion, Na-ion, Zr-air), and [2,3,4,5,6,7,8,9,10,11,12,13,14]. The production of biomass-based materials usually involves converting biomass into carbon-rich biochar through pyrolysis [15].

Biochar is a carbon-rich material produced by heating biomass in the absence of oxygen and is characterized by its remarkable porosity and large specific surface area. These attributes make it highly suitable for catalytic systems, where efficient interactions between the catalyst and the target substrate are critical for enhancing reaction performance [16]. Subsequently, it has been reported that biochar can act as catalyst support that can enhance catalytic activity by increasing the surface area and providing close contact with the target substrate through adsorption. Furthermore, its high electron conductivity can also inhibit electron recombination, which is useful in photocatalysis that has high recombination rates [2,17,18]. This dual functionality positions biochar as a key material in advanced catalytic and photocatalytic processes.

Indonesia, as the world’s largest producer of palm oil, accounts for 59% of the global production. This dominant role in the palm oil industry has led to the generation of significant volumes of biomass waste. Among the primary byproducts of palm oil production are empty fruit bunches (EFBs), which are left behind after the extraction of palm oil from full fruit bunches (FFBs). On average, processing one ton of FFBs yields approximately 254.7 kg of EFBs [19]. In 2023 alone, Indonesia produced an estimated 47 million tons of palm fruit, resulting in the generation of approximately 12 million tons of EFBs. This substantial amount of waste presents an opportunity for their innovative utilization. Owing to the large abundance and high lignocellulose content, EFBs hold significant promise as a raw material for biochar production. Converting EFBs into biochar not only helps address the environmental challenges associated with biomass waste but also offers a sustainable pathway for creating valuable materials with industrial and environmental applications [20].

In this study, we developed a straightforward and scalable approach to synthesizing biochar from EFB fibers through the pyrolysis process. The pyrolysis was conducted at various temperatures (i.e., 500, 800, and 1000 °C) to examine the effects of temperature on the structure and composition of the resulting biochar. Instrumental characterizations were performed to understand the transformations that occur during pyrolysis. Infrared spectroscopy was employed to identify functional groups and chemical changes, while Raman spectroscopy provided detailed insights into the structural evolution of the carbonaceous material. The Raman spectra were subsequently deconvoluted to analyze the degree of graphitization and the relative content of disordered and ordered carbon structures. These findings contribute to a growing body of knowledge on the conversion of Indonesian EFBs into biochar and highlight their potential for a wide range of applications, from catalysis to energy storage. Unlike many studies focusing on a single pyrolysis condition, our work comprehensively investigates biochar properties at three distinct pyrolysis temperatures. This allows us to elucidate the temperature-dependent structural evolution of biochar, including the transition from amorphous carbon to nano-crystalline graphite. Further, our study presents a novel contribution to biochar research by focusing on the underutilized resource of empty fruit bunches, developing scalable production techniques, enhancing biochar properties for diverse applications, and promoting environmental sustainability. We hope that this preliminary investigation sets the stage for further research aimed at optimizing biochar properties and exploring its integration into industrial processes.

## 2. Materials and Methods

### 2.1. Materials

EFB fibers were obtained from a local plantation in Middle Kalimantan, Indonesia. The aquadest used deionized water that was obtained from reverse osmosis.

### 2.2. Preparation of EFB and Biochars

The preparation of biochar from EFB fibers involved several steps. Initially, the raw EFB fibers were thoroughly washed with water to remove any dirt, debris, or contaminants that could interfere with the pyrolysis process. After washing, the fibers were dried in a laboratory oven set at 100 °C for 12 h to eliminate residual moisture, which is critical to achieving efficient pyrolysis and preventing unwanted reactions during heating. Once dried, the fibers were mechanically processed into a fine powder using a steel disc mill for 2 min. This milling step functions to increase the surface area, facilitating consistent heat transfer during the subsequent pyrolysis process.

The powdered EFB material was then subjected to pyrolysis in a high-temperature furnace under oxygen-free conditions to prevent combustion. The pyrolysis was conducted at three different temperatures, i.e., 500, 800, and 1000 °C, to study the effect of temperature on the properties of the resulting biochars. A ceramic crucible was used as the sample holder inside the furnace, which could hold around 50 g of EFB. The heating was carried out at a controlled rate of 3 °C/min to ensure gradual decomposition of the biomass and allow for nearly uniform heating temperature. Each pyrolysis run was maintained for 1 h at the target temperature to allow sufficient time to convert biomass into carbon-rich biochar. The yield percentages (%yield) were calculated using the following equation:

$$\%yield=\frac{m_{c}}{m_{0}}$$

where m_c_ is the total mass of the obtained biochar, and m_0_ is the starting mass of EFB before pyrolysis. The biochars obtained at the three different temperatures were labeled as C500, C800, and C1000, respectively, corresponding to the pyrolysis temperatures of 500, 800, and 1000 °C.

### 2.3. Material Characterization

Particle size analysis was done using Malvern Zetasizer Pro (ZSU 3200), which is capable of measuring size in the range of 0.3 nm to 10 µm. To ensure reliable and accurate measurements, the biochar samples were dispersed in aquadest and subjected to ultrasonication for 5 min prior to analysis. The sample-to-dispersant used was 1 g/L. This step was essential to break up any potential agglomerates and to achieve a uniform suspension of particles. N_2_ adsorption-desorption isotherms were measured at 77 K using a Quantachrome NOVA 2200E BET Surface Area Analyzer. Specific surface areas were determined using the Brunauer-Emmett-Teller (BET) method. Pore size and volume were calculated using the Barrett-Joyner-Halenda (BJH) method based on the adsorption branch. Before analysis, all samples were degassed under vacuum at 100 °C for 14 h to eliminate any adsorbed moisture. A slow degassing time at a relatively low temperature was used to ensure minimum changes or damage to the biochar samples, as discussed by Sigmund et al. (2017) [21]. Attenuated Total Reflectance-Fourier Transform Infrared (ATR-FTIR) spectroscopy was carried out using a Thermo Scientific Nicolet iS10 spectrometer equipped with a diamond crystal. This instrument facilitated spectral acquisition across a wavenumber range of 400 to 4000 cm⁻^1^. Raman spectroscopic analysis was conducted using a Thermo Scientific DXR2xi Raman microscope equipped with a 532 nm laser and a ×50 objective lens. The operating parameters include 5 mW laser power, 250 Hz exposure time, and 200 number of scans.

## 3. Results and Discussion

### 3.1. Variation of Mass

Upon calculations, we achieved a yield percentage of approximately 27, 23, and 20% for C500, C800, and C1000, respectively. This indicates that around 73–80% of the initial mass of the empty fruit bunch (EFB) was lost during pyrolysis, primarily due to the thermal decomposition of the lignocellulosic biomass. Such significant mass loss is attributed to the breakdown of cellulose, hemicellulose, and lignin, the three key constituents of lignocellulosic biomass, into various byproducts. Although these components decompose when pyrolyzed at 500 °C, complete carbonization of the biochar is not yet achieved, as further confirmed by the FTIR spectra. The observed mass reduction can be largely attributed to eliminating condensable and non-condensable gases, two of the three main products of biomass pyrolysis alongside char. Condensable gases include oxygenated hydrocarbons and moisture, while non-condensable gases encompass CO, CO_2_, and CH_4_, which are generated through decarboxylation, decarbonylation, and other thermal reactions [22]. A trend of decreasing yield with increasing pyrolysis temperature was also observed. This trend is related to the production of polycyclic aromatic hydrocarbons (PAHs) that are enhanced at temperatures over 700 °C. At the same time, gasification also occurs, explaining the continued decrease as pyrolysis temperature rises [23].

### 3.2. Particle Size and Surface Area Analysis

**Figure 1:**
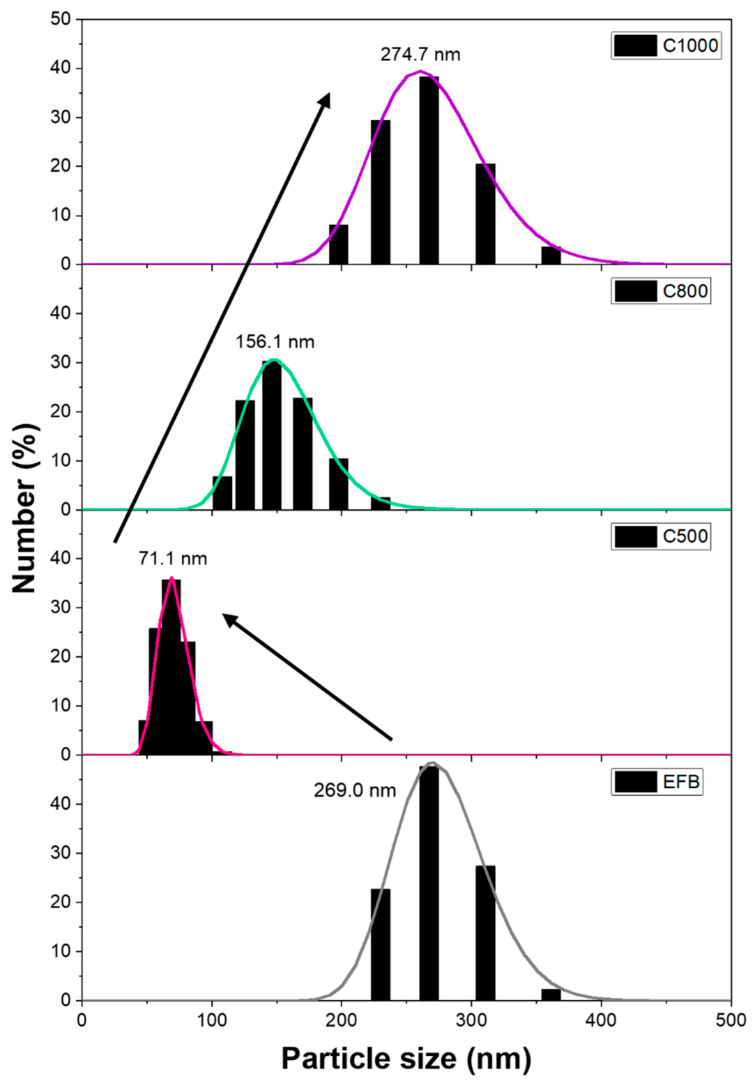
Particle size distribution of EFB fiber and biochar samples. The arrows indicate the particle change with increasing pyrolysis temperature from 0 to 1000 °C.

The particle size distribution, as seen in Figure 1, shows unimodal lognormal distributions with average particle sizes at 269.0, 71.1, 156.1, and 274.7 nm for EFB, C500, C800, and C1000, respectively. In comparison, particle size dropped significantly from untreated EFB to C500, followed by a gradual increase. These changes illustrate different steps inside the pyrolysis process. The first step includes the loss of moisture, which explains the decrease in size from 269.0 nm to 71.1 nm. The increase from C500 to C800 and C1000 is a result of prolonged exposure to heat. As the target pyrolysis temperature increases (from 500 °C to 800 °C and 1000 °C), the total time required to reach the final temperature increases because the heating progresses gradually at a fixed rate (3 °C/min). Thus, the samples spend more time under extreme heat. This extended heating duration influences biomass’s overall thermal decomposition behavior, particularly the water loss and the subsequent structural transformation of carbon materials. The prolonged exposure to heat promotes structural rearrangements, and amorphous carbon begins to transform into more ordered, graphitic domains, accompanied by eliminating residual heteroatoms and forming sp^2^-bonded carbon rings. This process, observed particularly at 1000 °C, contributes to the growth of nano-crystalline graphite with a higher degree of order, as evidenced by the Raman spectroscopy results. It has been reported that longer exposure to heat can influence both nucleation and agglomeration processes. Extended exposure can lead to the formation of more nucleated particles. The growth in particle size is largely driven by accelerated agglomeration. At high temperatures, the role of nucleation diminishes, and agglomeration becomes the dominant factor in particle growth [24].

Ultimately, based on changes in size and surface area, it was found that several different steps were at play to pyrolysis temperatures of 500 to 1000 °C, resulting in nonlinear trends that are subject to fluctuation. A more detailed explanation of the mechanism of biochar production is achieved when discussing the Raman spectroscopy results.

The BET and BJH methods were deployed to study the specific surface area, along with the pore size and volume of each biochar sample, as summarized in Table 1. It can be seen that the pore size of all three biochars was around 15 Å and did not relatively change with increasing pyrolysis temperatures. In contrast, the specific surface area and pore volume experienced an increase from C500 to C800, then decreased from C800 to C1000. It has been studied that increasing temperatures affect surface area and the development of pores. Higher pyrolysis temperatures generally promote the development of pores as gases are released during the thermal decomposition of biomass, which has also been observed in this study [25,26,27]. However, another study suggests that at particular temperatures, structural ordering happens, which involves the merging of pores, resulting in reduced surface area [28]. This explains the significantly lower specific surface area and pore volume of C500 compared to C800. The subsequent increase in surface area and pore volume from C800 to C1000 suggests that, beyond a certain temperature threshold, the release of additional gases and further carbonization reopen or create new pores, counteracting the effects of structural consolidation. This explains the nonlinear trend in specific surface area and pore volume across the temperature range.

### 3.3. FTIR Spectroscopy Analysis

**Figure 2:**
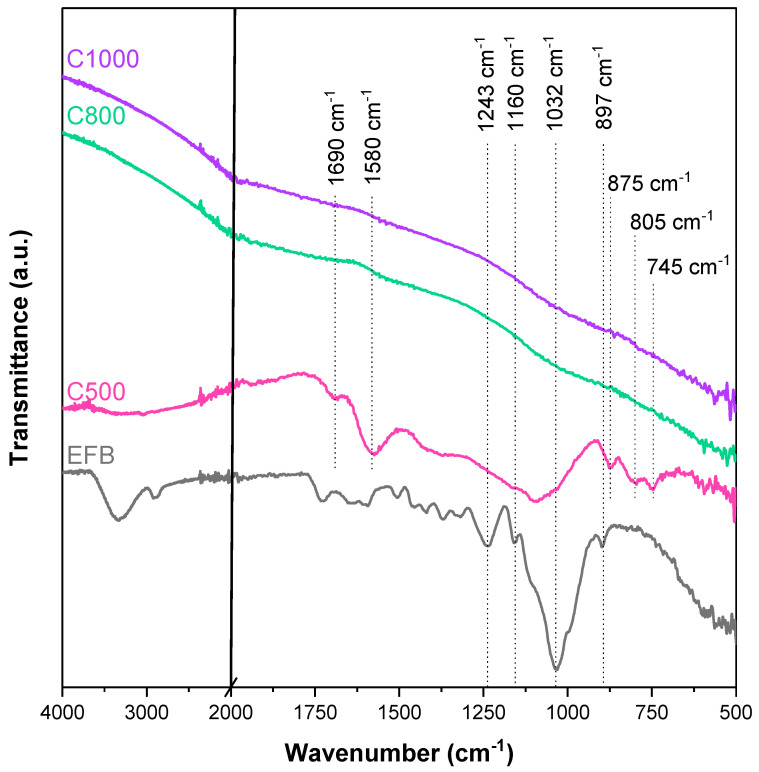
FTIR spectra of untreated EFB fiber and biochar samples.

Figure 2 presents the FTIR spectra of untreated and pyrolyzed EFB fibers at various pyrolysis temperatures, showcasing the structural changes that occur with increasing thermal treatment. The infrared (IR) absorbance profile of untreated EFB fibers is characteristic of lignocellulosic biomass, resembling spectra typically observed in materials such as agricultural shells, hulls, fibers, seeds, and wood. This similarity stems from the predominant presence of cellulose, hemicellulose, and lignin as the major components of the biomass [29,30,31,32]. In the spectra, three distinct peaks were seen at 3500–3000 cm^−1^, 2920 cm^−1^, and 2860 cm^−1^, corresponding to O−H bond stretching and asymmetric and symmetric stretching of C−H bonds, respectively. These peaks are indicative of the hydroxyl and aliphatic groups present in the lignocellulosic structure [33]. Moving to lower wavenumbers, the region between 1800 and 500 cm^−1^ shows a complex convolution of many different overlapping peaks, making it difficult to fully analyze. However, certain characteristic peaks can still be discerned. The peak at 1243 cm^−1^ can be attributed to C=O stretching or NH_2_ deformation of amino acids [33]. The peak at 1160 cm^−1^ is for asymmetric stretching of C−O−C, primarily associated with the polysaccharide components of cellulose and hemicellulose [34,35]. The noticeable peak at 1032 cm^−1^ is for the symmetric C−O stretching of cellulose, hemicellulose, and lignin [36,37]. Whereas the peak at 897 cm^−1^ is the β glycosidic bond of cellulose [33].

After pyrolysis at 500 °C, a significant change to the shape of the FTIR spectra was observed, signaling changes in structure and functional bonds. One of the most notable changes was the disappearance of the O−H stretching peak, which suggests that dehydration of the fibers occurred during this stage of thermal treatment [31]. This is consistent with the known decomposition behavior of lignocellulosic biomass, where both cellulose and hemicellulose undergo carbonization as they decompose, beginning at approximately 200 °C for hemicellulose and 327 °C for cellulose [38]. Additionally, new peaks emerged in the FTIR spectra, namely at 1690 cm^−1^ (C=O stretching for phenolic esters), 1580 cm^−1^ (aromatic C=C stretching), along with triple peaks at 875, 805, and 745 cm^−1^ (C−H bending for aromatic out-of-plane deformation), which indicates the formation of aromatic compounds. These observations confirm that the pyrolysis process at 500 °C facilitated significant carbonization, including the development of condensed aromatic compounds [37].

**Figure 3:**
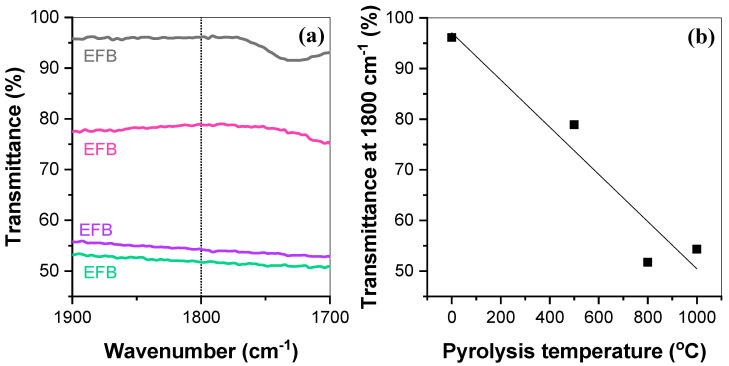
(**a**) The difference in baseline of EFB fiber and biochar samples at 1900–1700 cm^−1^. (**b**) The percent transmittance at 1800 cm^−1^ with increasing pyrolysis temperatures.

Figure 3 depicts the difference in the baseline of EFB fiber and biochar samples and the percent transmittance at 1800 cm^−1^ with increasing pyrolysis temperatures. At higher pyrolysis temperatures, specifically 800 °C and 1000 °C, the FTIR spectra produced a flat, tilted curve typically exhibited by carbonized materials with condensed aromatic structures [36,37,39]. This occurs due to low-energy electron excitations in graphitic domains, which reduce vibrational modes and diminish absorption features in the FTIR spectrum [39]. Further analysis of the functional groups was unfeasible due to this phenomenon. While FTIR spectroscopy is inherently limited in identifying crystalline carbon allotropes due to weakening vibrational signals with increased graphitization, we utilized the baseline elevation at specific transmittance values (e.g., 1800 cm⁻^1^) as a proxy to assess relative carbonization trends. A higher baseline corresponds to greater carbonization and aromaticity, providing a relative comparison rather than precise quantification. The transmittance value for biochar samples at 1800 cm⁻^1^ should be close to 100%, as demonstrated by the EFB spectra. However, it can be seen that transmittance is significantly lower than 100% for all three pyrolyzed samples, which is the result of the tilt curve caused by increased carbonization. The decreasing transmittance with increasing pyrolysis temperatures in Figure 3b illustrates that the degree of carbonization increases with pyrolysis temperature.

### 3.4. Raman Spectroscopy Analysis

**Figure 4:**
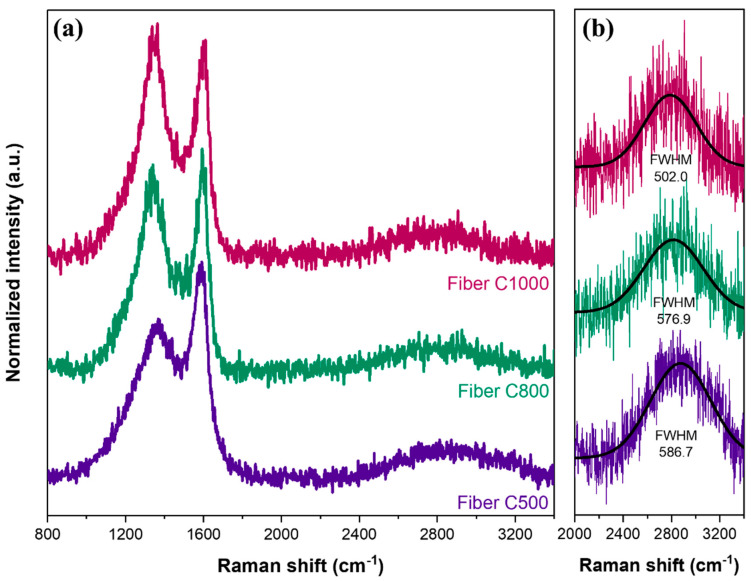
(**a**) The full Raman spectra and (**b**) the fitted peak at ~2900 cm^−1^ of biochar samples.

Figure 4a shows the Raman spectra of carbon samples pyrolyzed at different temperatures. In general, the shape of the obtained curves resembles a carbonaceous material, with two distinct peaks at approximately 1350 and 1600 cm^−1^, which is commonly referred to as the D (“defect” or “disorder”) band and the G (“graphite”) band, respectively. Analyzing the width, intensity, and peak positions of the D and G bands allows for the differentiation of carbon allotropes and provides insight into the structural properties of the materials. The broad D and G bands observed in the spectra are characteristic of sp^2^-dominated amorphous carbon, such as those found in glassy carbon, chars, activated carbons, and carbon black [40,41,42,43]. A noticeable transformation in the Raman spectra is observed as the pyrolysis temperature increases [44]. There is a shift in peak position (Figure 4b) with pyrolysis temperature. However, no shifting in the D band was found after deconvolution. Instead of shifting, the D-to-G intensity ratio has increased with the rise in temperatures, which has been explained in more detail in the subsequent section. Regardless, this temperature-dependent evolution underscores the influence of pyrolysis conditions on the structural properties of carbon materials.

**Figure 5:**
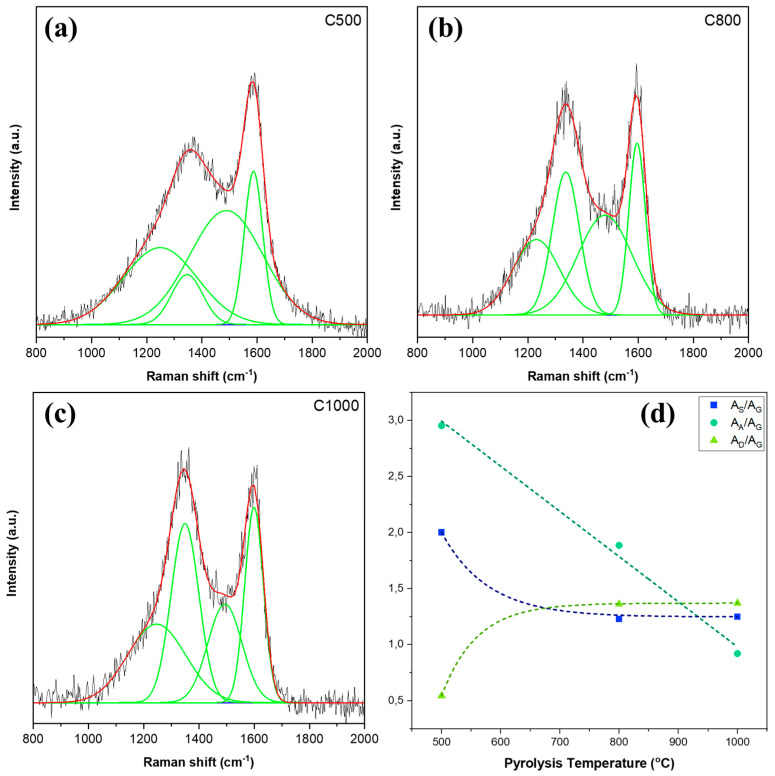
(**a**–**c**) Deconvolution of the Raman spectra of biochar samples into S, D, A, and G peaks, respectively (green) and the total summed peaks (red). (**d**) The peak area changes of S (blue), A (green), and D (light green) relative to the G peak with increasing pyrolysis temperature.

The Raman spectra can be further analyzed through deconvolution, as seen in Figure 5a–c. Here, we fitted four peaks at approximately 1250, 1350, 1500, and 1600 cm^−1^ for the S, D, A, and G bands, respectively. Each of these bands provides critical insights into the structural characteristics of the carbon materials. The G band, located at around 1600 cm⁻^1^, corresponds to the in-plane vibration of sp^2^-bonded carbon atoms. This band signifies the presence of ordered graphitic domains, reflecting the degree of structural ordering within the carbon sample. However, a higher G peak will not always correspond to a well-ordered structure, as will be discussed in more detail later in this section [45]. Meanwhile, the A band, centered near 1500 cm⁻^1^, denotes the amorphous domains of the sample relating to irregular structures. Smith et al. (2016) predicted that the A band might be linked to vibrations dominated by Kekulé structures, the out-of-plane deformations occurring around defects, and oxygen heteroatom defects [46]. Next, the D band, appearing at approximately 1350 cm⁻^1^, results from breathing modes of sp^2^ at grain boundaries and defect points, hence the relation with defects and disorder [40]. Finally, the S (shoulder) band, found around 1250 cm⁻^1^, is linked to breathing and Kekulé vibrations of small polyaromatic hydrocarbons (PAHs), highlighting the presence of these molecular structures in the sample [46].

Through this detailed deconvolution analysis, the complex interplay of ordered and disordered carbon structures within the pyrolyzed samples can be better understood; comparing the different peaks offers a deeper insight into the transformation of carbonaceous materials after pyrolysis at varied temperatures. Given the broad nature of the Raman peaks, analyzing the area ratios provides a more reliable method than intensity ratios for understanding the structural changes in the samples. However, consistent trends between the intensity and area ratios were observed (Figure S2), indicating that the use of the area ratio does not significantly affect our results. All the deconvoluted peaks were normalized against the G band for comparative analysis, as shown in Figure 5d. The area ratio of the A band to the G band (A_A_/A_G_) shows a negative linear trend with increasing pyrolysis temperature. This indicates that the amorphous species within our sample decreases, indicating the transition from disordered to more ordered carbon structures as pyrolysis proceeds. This reduction in amorphous carbon correlates with the increased graphitization observed at higher temperatures.

At the same time, the area ratio of the S-band to the G band (A_S_/A_G_) experienced an exponential decay. The most significant reduction occurs between 500 °C and 800 °C, suggesting the rapid decomposition of small polyaromatic hydrocarbons (PAHs) within this temperature range. After 800 °C, the ratio value starts to saturate, indicating that most of the small PAHs have already decomposed and that the structural changes are nearing completion. This change aligns with the observed trend of A_A_/A_G_ and the increased graphitization observed at higher temperatures.

The increase in the ratio of the D band to the G band A_D_/A_G_ normally signals the increase of disorder within our sample. However, in our case, the observed trend indicates that the changes observed are a result of the transition from amorphous carbon to nano-crystalline graphite, as discussed by Ferrari and Robertson (2000). They explained that, in highly amorphous carbon systems, such as our biochar pyrolyzed at 500 °C, there are only a few rings present in the carbon structure. Since the D band arises from the sp^2^ breathing mode of rings, while the G band results from the vibration of any sp^2^ carbons, the D-to-G ratio will be lower due to the small number of rings, and the rise of A_D/_A_G_ demonstrates an ordering process as more rings are produced. Figure 5d shows that A_D/_A_G_ increases up to a certain point and then starts to saturate after 800 °C. This saturation suggests that the preliminary stage of converting amorphous carbon into nano-crystalline graphite has been completed. Beyond this temperature, the graphitic structure becomes more developed. Further pyrolysis at higher temperatures should decrease the D-to-G ratio as the monocrystalline structure is converted into polycrystalline, and graphite is formed. In this phase, the expected trend is that a lower D-to-G ratio will indicate a higher proportion of graphitic content, reflecting the increased crystallinity and structural order of the carbon material [47]. These findings are consistent with the trends of A_S/_A_G_ and A_A/_A_G_, as well as the changes in particle size and surface area, which have previously been discussed. Furthermore, this also follows the general understanding of carbon transformation during pyrolysis, providing insight into the evolution of EFB fibers at different pyrolysis temperatures.

Additionally, a broad peak was also observed at 2400–3400 cm^−1^, as displayed in the inset of Figure 4b, which is another effect of the transition from amorphous carbon to nano-crystalline graphite. The peaks for all samples were fitted, and the FWHM was calculated. The increase in FWHM as pyrolysis temperature goes up signals the emergence of crystalline graphite [47]. It is noteworthy that Raman’s theory used to model carbon structures, including the relationship between the D-band and G-band intensities, is primarily based on crystalline carbon materials. Consequently, its direct application to materials with significant structural defects, such as biochar, requires careful consideration. In our study, the D-to-G band intensity ratio (I_D_/I_G_) was used as a relative indicator to track the progression of carbonization and the formation of nano-crystalline graphite [48,49]. While we agree that this ratio does not provide absolute quantification of graphitization in defective carbon systems, it remains a useful tool for identifying trends in structural transformation with increasing pyrolysis temperatures.

### 3.5. Crystallite Size Analysis

At lower pyrolysis temperatures, biochar retains significant amorphous carbon with disordered structures. XRD tends to exhibit broad, overlapping peaks for such materials, making it less sensitive to distinguishing between amorphous carbon and early-stage nano-crystalline graphite. On the other hand, Raman spectroscopy is highly sensitive to graphitization and structural transitions. Further, the D-band and G-band allowed us to identify the formation of nano-crystalline graphite and quantify the disorder through the I(D)/I(G) ratio. Based on the above findings, we estimate that the biochar produced at 1000 °C (C1000) has undergone nearly complete carbonization and is beginning to exhibit an increase in crystallite size (La). To quantify La, we employed a modified version of the equation derived from the Tuninstra-Koenig law, which accounts for the energy dependence of the Raman spectra. The equation is as follows:

$$L_{a}=\frac{4.4}{\frac{I_{D}}{I_{G}}}\times {\left(\frac{2.41}{E_{L}}\right)}^{4}$$

where L_a_ represents the crystallite size in nanometers (nm), I_D_/I_G_ is the intensity ratio of the G and D bands, and E_L_ is the laser excitation energy in electron volts (eV), which in our case is 2.33 eV (532 nm) [50,51,52]. From this equation, we found that the crystallite size of C1000 is approximately 5.47 nm. This relatively small crystallite size indicates a high defect density, which is advantageous for certain applications such as catalysis and adsorption. Defects in carbon materials often enhance their catalytic activity by providing active sites and facilitating the adsorption of reactants [53]. However, the high defect density also results in reduced electrical conductivity, which could hinder the performance of C1000 in electrocatalytic applications [54].

**Figure 6:**
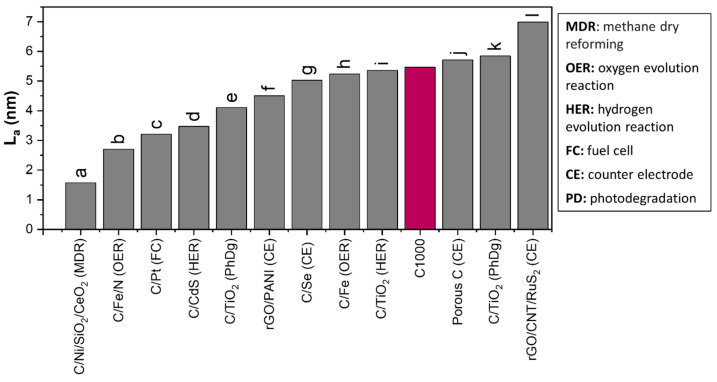
Comparison of the L_a_ from other studies that also utilize similar, mostly amorphous, carbons for various applications, i.e., (a) [57], (b) [58], (c) [59], (d) [60], (e) [61], (f) [53], (g) [62], (h) [63], (i) [64], (j) [65], (k) [66], and (l) [67].

Additional modifications may be necessary to balance defect density and conductivity and optimize the utility of C1000 biochar. Potential strategies include doping with heteroatoms, exfoliation, oxidation, surface functionalization, and physical and chemical activation [49,55,56]. Additionally, further modifications to give ferromagnetic properties have also been explored, as demonstrated by Lyu et al. (2024) through metal-nitrogen doping, highlighting the versatility of carbon-based materials [56]. Alternatively, the current properties of C1000 make it a promising candidate for use as a catalyst support. Its high surface area and defect-rich structure could enhance the performance of supported catalysts in various applications, such as solar cell counter electrodes, electrocatalysts, and photocatalysts, as demonstrated in Figure 6, which compares the crystallite size of our C1000 biochar with other carbon-based catalyst supports [53,57,58,59,60,61,62,63,64,65,66,67]. All the cited reports utilize carbonaceous materials that have wide overlapping D and G peaks, like the samples discussed in this study. This comparison highlights the competitive edge of C1000 biochar in terms of its nanoscale structure, suggesting potential for integration into advanced catalytic systems. Further research and optimization could pave the way for its application in sustainable and high-performance catalytic processes. It should be noted that the presented La is only a rough estimate and is used here to better compare with other studies.

## 4. Conclusions

Based on FTIR spectroscopy analysis, we observed that biochar produced at 500 °C had not yet undergone complete carbonization. This contrasts with the biochar produced at 800 °C and 1000 °C, which showed characteristics of advanced carbonization stages. Raman spectroscopy further supports this finding, as the D-to-G ratio increased significantly from 500 °C to 800 °C, indicating a structural transition from amorphous carbon to nano-crystalline graphite. The observed reduction in amorphous carbon content and the decomposition of small polyaromatic hydrocarbons (PAHs) with increasing pyrolysis temperature also corroborate this structural evolution. Furthermore, crystallite size calculations suggest that the biochar produced at 1000 °C possesses a high defect density, which makes it a promising candidate for use in various catalyst support applications. The defect-rich structure can enhance catalytic activity, while high porosity can assist in adsorption. These properties provide a foundation for potential uses in advanced catalytic systems, such as photocatalysts, electrocatalysts, or solar cell counter electrodes. These findings highlight the adaptability of biochar synthesized at different pyrolysis temperatures for diverse industrial applications, particularly in catalysis and adsorption. Apart from application studies, further research should include the characterization of carbon pyrolyzed at higher temperatures that aim to produce well-ordered graphite with higher purity. Other methods should also be explored, such as microwave-assisted pyrolysis, flash joule heating, and ultrasonication [68]. While also considering the cost and scalability of the process.

## Figures and Tables

**Table 1:** Comparative Specific surface area, pore size, and volume of biochar samples estimated from the BET-BJH analysis. The adsorption isotherms are shown in Figure S1.

Sample	Specific Surface Area (m^2^/g)	Pore Volume (cm^3^/g)	Pore Size (Å)
C500	590	0.9	10.1
C800	1060	1.6	15.3
C1000	940	1.1	12.4

## Data Availability

The data presented in this study are available on reasonable request from the corresponding author.

## Supplementary Materials

The following supporting information can be downloaded at: https://www.mdpi.com/article/10.3390/nano15010050/s1, Figure S1: Nitrogen sorption isotherm of biochar pyrolyzed at 500 °C, 800 °C, and 1000 °C; Figure S2: The peak area changes relative to the G peak with increasing pyrolysis temperature.
