# Supplementary material for: Utilizing Indonesian Empty Palm Fruit Bunches: Biochar Synthesis via Temperatures Dependent Pyrolysis
Source: Nanomaterials (Basel). 2024 Dec 31;15(1):50. doi: 10.3390/nano15010050 (PMC11723393; doi:10.3390/nano15010050)
Supplement: Supplementary file 1 [file nanomaterials-15-00050-s001.zip › nanomaterials-3372027-supplementary.pdf]

*Supporting Information*

# Utilizing Indonesian Empty Palm Fruit Bunches: Biochar Synthesis via Temperatures Dependent Pyrolysis

**Fairuz Gianirfan Nugroho [1,†], Abu Saad Ansari [1,†], Nurul Taufiqu Rochman [2], Shubhangi Satish Khadtare [3], Vijaya Gopalan Sree [4], Nabeen K. Shrestha [4], Afina Faza Hafiyyan [1], Hyunsik Im [4] and Abu Talha Aqueel Ahmed [4,*]**

[1] Center of Excellence Applied Nanotechnology, Nano Center Indonesia, Puspiptek, South Tangerang 15314, Banten, Indonesia; fairuz.gianirfan@gmail.com (F.G.N.); saad@nano.or.id (A.S.A.)
[2] Research Center for Advanced Material, National Research and Innovation Agency (BRIN), Puspiptek, South Tangerang 15314, Banten, Indonesia; nurul@nano.or.id
[3] Department of Electronic Engineering, Hanyang University, Seoul 04763, Republic of Korea
[4] Division of Physics and Semiconductor, Dongguk University, Seoul 04620, Republic of Korea
* Correspondence: abutalha.aa@dongguk.edu
† These authors contributed equally to this work.

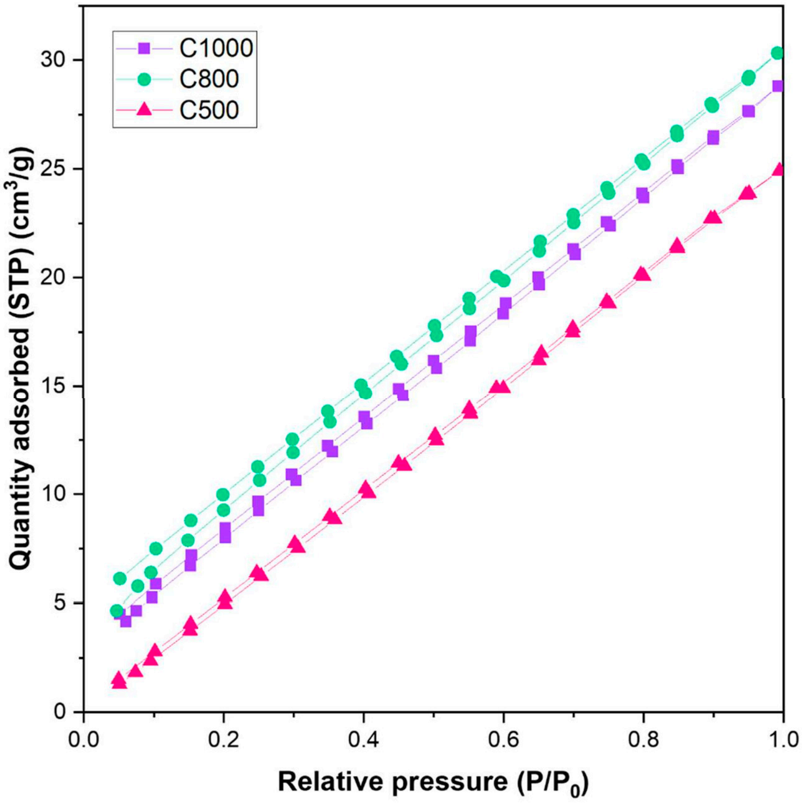


**Figure S1.** Nitrogen sorption isotherm of biochar pyrolyzed at 500 °C, 800 °C, and 1000 °C.

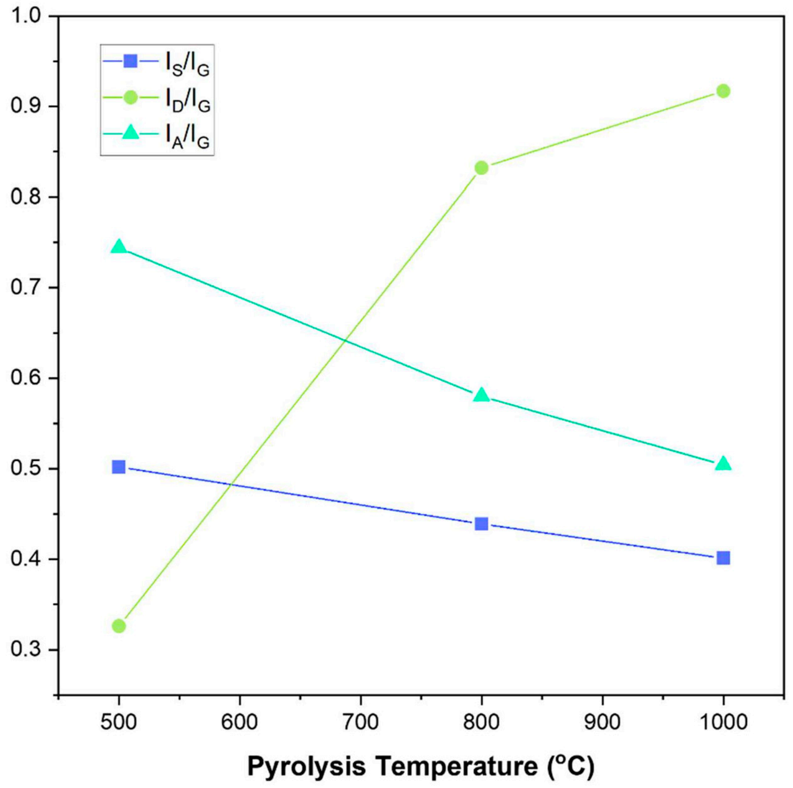


**Figure S2.** The peak area changes relative to the G peak with increasing pyrolysis temperature.
